# Neuronal *Lhx1* expression is regulated by DNMT1-dependent modulation of histone marks

**DOI:** 10.1080/15592294.2020.1767372

**Published:** 2020-05-22

**Authors:** Judit Symmank, Cathrin Bayer, Julia Reichard, Daniel Pensold, Geraldine Zimmer-Bensch

**Affiliations:** a Institute for Human Genetics, Am Klinikum 1, University Hospital Jena, Jena, Germany; b Polyclinic for Orthodontics, Leutragraben 3, University Hospital Jena, Jena, Germany; c Department of Functional Epigenetics in the Animal Model, Institute for Biology II, Worringerweg 3, RWTH Aachen University, Aachen, Germany; d Research Training Group 2416 MultiSenses, MultiScales, RWTH Aachen University, Aachen, Germany

**Keywords:** Interneuron development, LHX1, DNMT1, histone methylation, histone acetylation, epigenetic network

## Abstract

Apart from the conventional view of repressive promoter methylation, the DNA methyltransferase 1 (DNMT1) was recently described to modulate gene expression through a variety of interactions with diverse epigenetic key players. We here investigated the DNMT1-dependent transcriptional control of the homeobox transcription factor LHX1, which we previously identified as an important regulator in cortical interneuron development. We found that LHX1 expression in embryonic interneurons originating in the embryonic pre-optic area (POA) is regulated by non-canonic DNMT1 function. Analysis of histone methylation and acetylation revealed that both epigenetic modifications seem to be implicated in the control of *Lhx1* gene activity and that DNMT1 contributes to their proper establishment. This study sheds further light on the regulatory network of cortical interneuron development including the complex interplay of epigenetic mechanisms.

## Introduction

An increasing number of studies challenged the textbook model of repressive DNA methylation that is catalysed by DNA methyltransferases (DNMTs). The identification of the diverse genomic locations that can be methylated like enhancer and intragenic loci in addition to promoter regions has led to new findings for functional implications of DNA methylation like alternative splicing and promoter choice [1–3]. Moreover, in contrast to the traditional view of DNA methylation preventing the binding of transcription factors, numerous reports indicated that DNA methylation signatures can even serve as binding motifs for particular factors, thereby mediating methylation-dependent biological processes [4]. Apart from this, DNA methylation can instruct histone-modifying complexes (HMCs) and *vice versa* [5–8]. In addition, DNMTs act on histone modifications by transcriptional control over genes encoding for proteins implicated in HMCs or by interacting with protein complexes independent of their DNA methylating activity [9–12]. This diversity of actions requires detailed investigations to decipher the functional implications of distinct epigenetic mechanisms in directing cell- and stage-specific differentiation and maturation programmes, and to reveal causes for dysfunctions in related diseases.

Alterations in epigenetic signatures or the function of epigenetic key players in neurons were reported to contribute to the pathophysiology of diverse neurological diseases and psychiatric disorders [13,14]. Changed *Dnmt1* expression was observed *post-mortem* in inhibitory cortical interneurons of schizophrenia patients, which is suggested to be associated with altered expression of GABA-related transcripts [15,16]. By shaping the response of excitatory neurons through inhibitory actions, GABAergic interneurons are essential key players in cortical information processing. For this, it is not surprising that numerous neuropsychiatric diseases like schizophrenia, epilepsy, and autism involve defects in GABAergic interneuron function [17–20], which are suggested to be in part developmental in their origin [18–20]. In support of this, prenatal stress alters DNA methylation networks in inhibitory cortical interneurons during development, which elicits a schizophrenia-like phenotype in offspring [21–23]. However, little is known so far about the stage- and context-specific effects of epigenetic transcriptional regulation during cortical interneuron development, which is a highly complex process [24]. A major step embraces the long-range migration of post-mitotic interneurons from their sites of origin in the basal telencephalon towards cortical target areas [24–27]. This requires comprehensive control over cytoskeletal remodelling to achieve successful migration, prerequisite for the correct number of cortical interneurons in the diverse cortical regions [26–28]. The strict regulation of cell survival during the different developmental steps is likewise critical for proper interneuron numbers in adults [26,27,29].

We have recently reported that the DNA methyltransferase 1 (DNMT1) orchestrates the post-mitotic maturation of POA-derived cortical interneurons by promoting their migratory morphology and survival in part through the modulation of *Pak6* expression [27]. Of note, we found that *Pak6* transcription is not regulated by DNMT1-dependent DNA methylation [12,27], but non-canonically through interactions of DNMT1 with histone methylating enzymes [12].

Apart from DNMT1, we identified the LIM-homeobox transcription factor LHX1 as crucial transcriptional regulator of POA-derived inhibitory interneuron development [26]. LHX1 modulates the expression of major guidance receptors in migrating interneurons facilitating their tangential and radial migration through the basal telencephalon and the developing cortex, respectively [26]. Alike DNMT1, LHX1 acts on interneuron survival by controlling gene expression of genes like *Bcl2* or *Bcl6* [26]. Thereby, the expression of *Lhx1* is restricted to early post-mitotic stages and timed *Lhx1* silencing is critical for the proper regulation of interneuron survival and migration during development [26]. To this end, we here investigated whether and how *Lhx1* expression is controlled by DNMT1 as a potential upstream regulator.

We identified *Lhx1* expression to be controlled by non-canonical DNMT1 activity. Besides evidence for a DNMT1-dependent bivalent regulation through H3K4 and H3K27 trimethylation, our data propose a contribution of DNMT1-mediated histone acetylation and deacetylation to the regulation of *Lhx1* expression. This study emphasizes the complexity of epigenetic networks in transcriptional control of key players relevant for cortical interneuron development.

## Results

### DNMT1  regulates the expression of Lhx1 non-canonically

**Figure 1.**
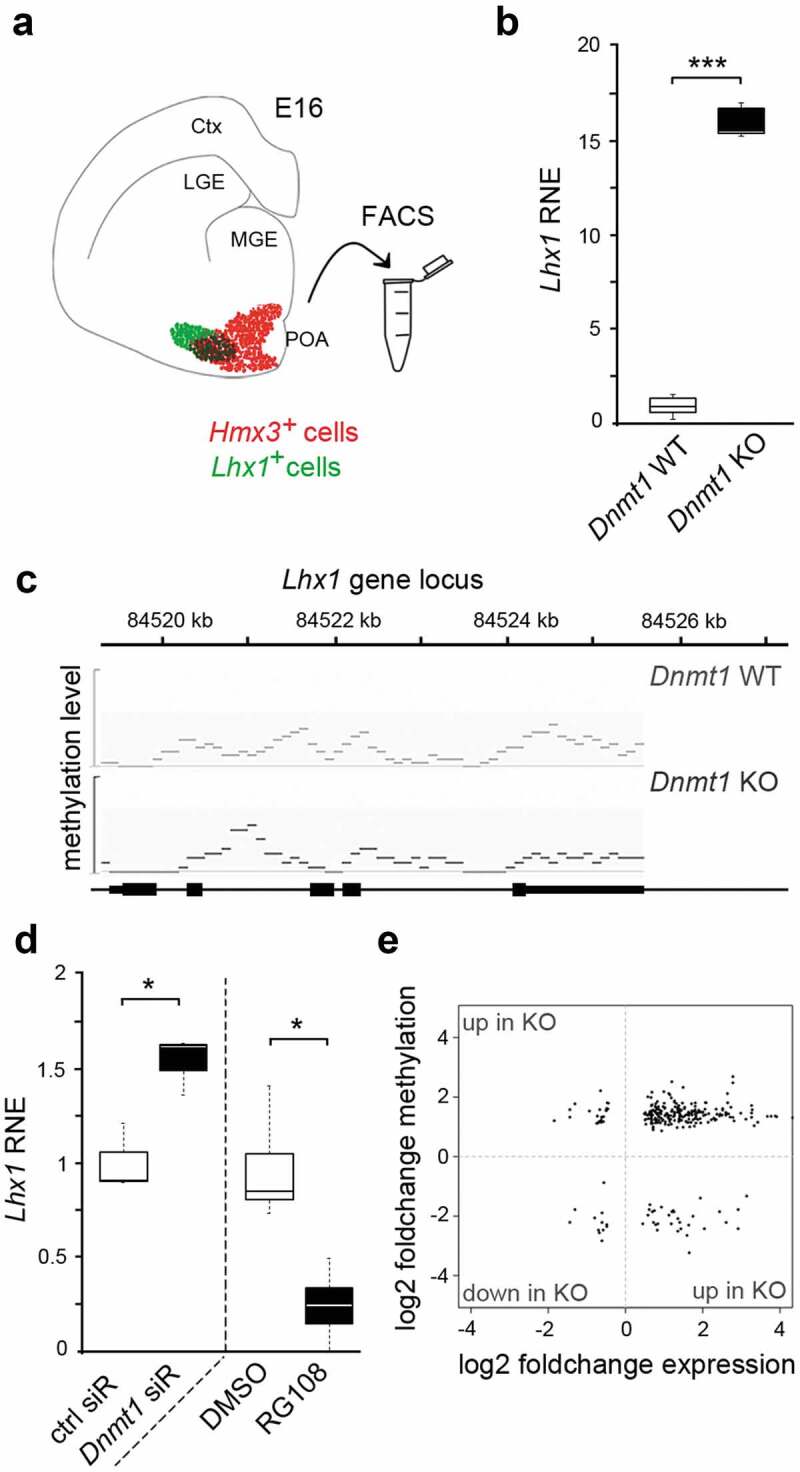
*Lhx1* transcription is controlled by DNMT1, but not through its DNA methylation activity

In the embryonic mouse brain, *Lhx1* is very restrictively expressed in the mantle zone of the embryonic POA, partially overlapping with the local and post-mitotic expression of the transcription factor HMX3 (Figure 1(a) [26]). We have previously shown that LHX1-dependent transcriptional control is of great relevance for the survival and migration regulation in post-mitotic HMX3-positive cortical interneurons originating in the POA [26]. For this immature interneuron subset, we further identified DNMT1 to be essential for orchestrating stage-specific gene expression [27]. Hence, in this study, we first aimed to investigate whether DNMT1 controls the expression of *Lhx1*.

(a) Schematic illustration of a mouse brain coronal section at embryonic day (E) 16 depicting *Hmx3*- (red) and *Lhx1*-positive (green) cells located in the preoptic area. (b) Quantitative Real-Time PCR displays *Lhx1* expression in FACS-enriched *Hmx3-Cre/tdTomato/Dnmt1 loxP^2^* cells (*Dnmt1* KO) compared to *Hmx3-Cre/tdTomato/Dnmt1* wild-type cells (*Dnmt1* WT) of E16 mice. (c) MeDIP sequencing analysis of the *Lhx1* gene locus of *Dnmt1* WT and *Dnmt1* KO mice. (d)Quantitative Real-Time PCR displays the *Lhx1* expression in N2a cells treated either with *Dnmt1* siRNA or RG108 in comparison to control siRNA- or DMSO-treated N2a cells. (e) Scatter Plot of genes indicating changes in their methylation and expression levels between *Hmx3-Cre/tdTomato/Dnmt1 loxP^2^* cells and *Hmx3-Cre/tdTomato/Dnmt1* wild-type cells of E16 mice. *P < 0.05; ***P < 0.001; Student’s *t*-test. Ctrl, control; ctx, cortex; LGE, lateral ganglionic eminence (GE); MGE, medial GE; POA, preoptic area; RNE, relative normalized expression.

To this end, we checked for changes in the expression levels of *Lhx1* in FACS-enriched *Hmx3-Cre/tdTomato/Dnmt1* control and *Hmx3-Cre/tdTomato/Dnmt1* interneurons isolated from the POA of mouse embryos at embryonic day (E) 16, at the peak of POA interneuron migration (Figure 1(a)). Indeed, we detected a highly elevated *Lhx1* expression in *Dnmt1* knockout (KO) cells, which points to a DNMT1-mediated repression of *Lhx1* in wild-type interneurons (Figure 1(b)).

To control whether these transcriptional changes correlate with alterations in DNA methylation levels, we screened the MeDIP-sequencing data performed with equally aged embryonic FACS-enriched control and *Dnmt1* KO cells (E16), published previously in Pensold *et al*. [27]. However, the *Lhx1* gene locus did not show any significant alterations in the DNA methylation levels between the two genotypes (Figure 1(c)). Apart from this, the RNA-sequencing and MeDIP-sequencing dataset of FACS-enriched *Dnmt1* KO and control cells (E16) [27] did not reveal altered methylation and expression levels of potential regulators of *Lhx1* in *Dnmt1*-deficient interneurons. In agreement with the expression and DNA methylation analysis of POA-derived embryonic interneurons, elevated levels of *Lhx1* expression were also detected upon *Dnmt1* siRNA application in Neuro2a (N2a) cells, a cell culture model already applied in a previous study [12]. Of note, this increase in *Lhx1* expression was not observed upon treatment with RG108, an inhibitor of DNA methylation (Figure 1(d)), which even leads to a significant decrease putatively by eliciting secondary effects. Together, our data suggest a DNA methylation-independent repression of *Lhx1* by DNMT1.

In general, direct effects of repressive DNMT1-dependent DNA methylation appear to play a rather subordinate role during embryonic development of POA-derived interneurons. First, by MeDIP and RNA sequencing we identified a non-significant overlap of genes displaying both, changes in the methylation and expression profiles in *Dnmt1*-deficient *Hmx3-Cre/tdTomato* compared to control cells [12]. Second, among the overlapping genes, we identified only very few genes displaying reduced DNA methylation and increased expression in the *Dnmt1*-deficient cells (Figure 1(e), lower right quadrant), which would be consistent with the canonical function of DNMT1 performing repressive DNA methylation in controls. In turn, most genes were elevated in expression and displayed at the same time increased levels of DNA methylation (Figure 1(e), upper right quadrant), pointing to secondary or indirect effects caused by *Dnmt1* deletion. This is consistent with the emerging new functional implications of DNA methylation being far more complex than just leading to gene repression. DNA methylation is described to mediate alternative splicing and promoter choice [1–3] and can even lead to the formation of binding motifs for particular factors that upon binding drive the transcription of particular genes [4].

In sum, our data so far suggest that DNMT1 exerts transcriptional control over *Lhx1* in embryonic POA-derived interneurons, but rather independent of direct DNA methylation of the *Lhx1* gene locus or gene loci encoding for known *Lhx1* regulators.

### Dnmt1 *deficiency resulted in altered H3K4me3 and H3K27me3 levels at the* Lhx1 *gene locus*

In addition to its DNA methylating activity, we have recently reported that non-canonical functions of DNMT1, such as a crosstalk with histone-modifying enzymes, are involved in the transcriptional regulation in developing interneurons [12]. In detail, *Pak6* expression was found to be controlled through direct or indirect interactions of DNMT1 with EZH2, the core enzyme of the polycomb-repressor complex 2 (PRC2), catalysing repressive trimethylations at H3K27 [30,31].

**Figure 2.**
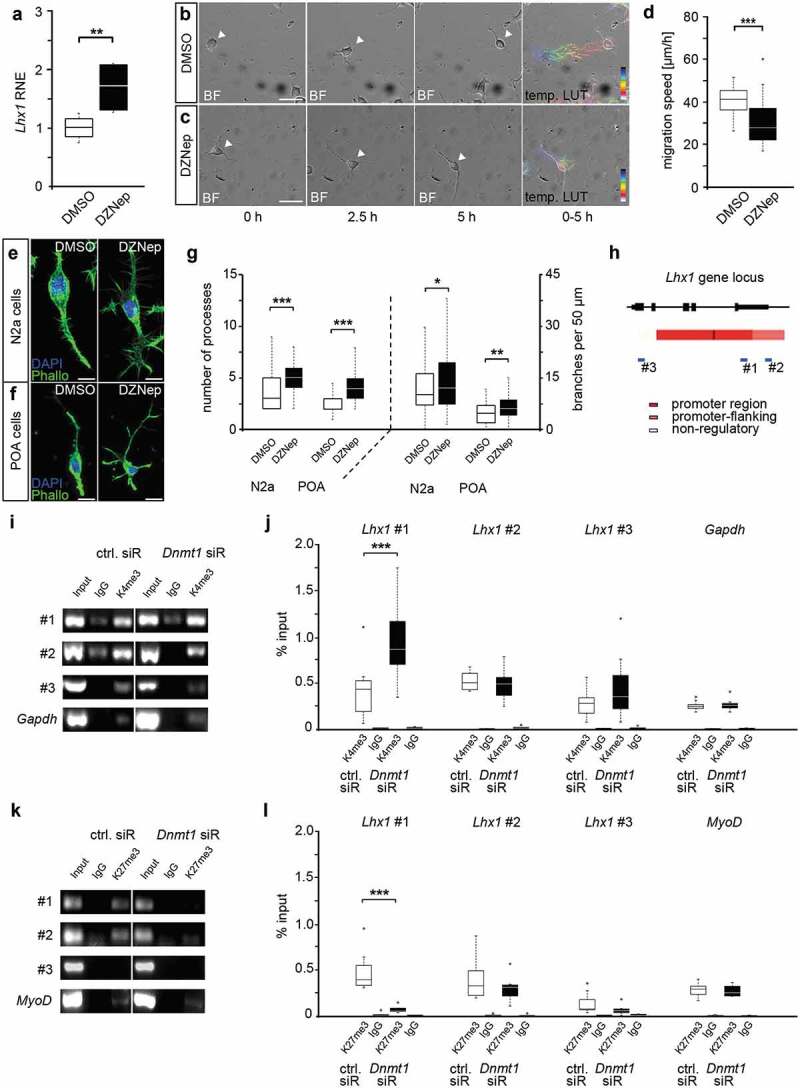
DNMT1-dependent modulation of repressive and permissive histone lysine trimethylation contributes to *Lhx1* expression control

To evaluate a potential implication of DNMT1-dependent modulation of repressive histone methylation in the transcriptional control of *Lhx1*, we analysed *Lhx1* expression in N2a cells upon treatment with 3-deazaneplanocin A (DZNep, Figure 2(a)), a potent inhibitor of the histone methyltransferase EZH2 [32,33]. We detected significantly elevated *Lhx1* expression levels in DZNep-treated N2a cells (Figure 2(a)), suggesting a role of histone methylation in repressing *Lhx1* transcription. As LHX1 was shown to influence POA cell migration [26], we next analysed whether DZNep-treatment affects the migratory potential of N2a cells. To this end, we monitored the migratory speed of N2a cells on matrigel, which was significantly decreased upon DZNep-treatment compared to control treatment with DMSO (Figure 2(b–d)). In line with that, we found DZNep-induced morphological alterations which could account for the reduced motility. By comparing the morphology of DZNep- and control-treated POA and N2a cells, we detected increased numbers of processes as well as higher numbers of branch points of the longest process of each cell (Figure 2(e–g)), which is indicative for the loss of the polarized migratory morphology.

(a) Quantitative Real-Time PCR shows *Lhx1* expression in N2a cells treated either with DZNep or DMSO as control. (b, c) Life cell imaging analysis of migrating N2a cells treated with DZNep and DMSO as control. Representative brightfield images of temporal sequences illustrating N2a cells and their migratory behaviour after treatment with DMSO (b) or DZNep (c) depicting frames at 0 h, 2.5 h, and 5 h. The white arrowhead points to the soma of the monitored cell. The last panel in (b) and (c) represents the colour-coded migratory distances within the analysed 5 h (temporal LUT), indicating the starting point (deep blue) and end (white) of the respective cell. (d) Quantification of migration speed of DMSO- and DZNep-treated N2a cells (in µm/h; n = 30 cells for each condition in three independent experiments). (e, f) Representative microphotographs of N2a (e) and POA cells (f) treated with DZNep (N2a cells n = 93; POA n = 69) in comparison to DMSO control (N2a cells n = 85; POA n = 52) with F-actin stained using phalloidin (green) and nucleus staining (DAPI, blue). The number of processes from cell soma and of branches from the longest process is shown in (g). (h) Schematic illustration according to UCSC genome browser displaying the *Lhx1* gene locus with promoter (red), promoter flanking (light red), and non-regulatory (white) sites. DNA primer positions (#1, #2, #3) for quantitative PCR are indicated with blue bars. (i–l) Targeted ChIP analysis showing the association of H3K4me3 (i, j) and H3K27me3 (k, l) to the primer positions #1–#3 at the *Lhx1* gene locus of N2a cells, treated with *Dnmt1* siRNA in comparison to control siRNA, and analysed by quantitative Real-Time PCR and normalized to input controls. The amount of non-specifically bound DNA is indicated to IgG controls. Scale bar: 50 µm in (b) and (c), 10 µm in (e) and (f). *P < 0.05; ***P < 0.001; Student’s *t*-test. BF: brightfield, Ctrl, control; Phallo, phalloidin., temp. LUT: temporal LUT; RNE, relative normalized expression.

We previously showed that DNMT1 negatively acts on permissive H3K4me3 levels and promotes the establishment of repressive trimethylation of H3K27 at the global level [12]. Here we investigated whether DNMT1 is required to prevent or promote the setup of permissive H3K4- or repressive H3K27-trimethylation marks at regulatory sites of the *Lhx1* gene locus, respectively. For this we performed targeted chromatin-immunoprecipitation (ChIP) with an H3K4me3- and an H3K27me3-specific antibody followed by qPCR with primers directed against regulatory and non-regulatory regions of the *Lhx1* locus in control and *Dnmt1* siRNA-treated N2a cells (Figure 2(h–l)). Compared to controls, we identified an enhanced association of H3K4me3 within the promoter region of the *Lhx1* gene locus in *Dnmt1*-depleted compared to control cells, whereas promoter-flanking and non-regulatory regions did not reveal detectable changes (Figure 2(h–j)). As *Gapdh* is often associated with H3K4me3 in neurons [34,35], it was used as a positive control. This suggests that DNMT1 negatively influences the establishment of permissive H3K4me3 marks at the *Lhx1* promoter region.

Many promoters are bivalently regulated by H3K4me3 and H3K27me3 [36–41], and we already showed a DNMT1-dependent establishment of H3K27me3 signatures in immature POA-derived interneurons and neuron-like N2a cells [12]. Consistent with the global reduction of H3K27me3 found upon *Dnmt1* depletion [12], H3K27me3 association was significantly diminished site-specifically at the promoter region of the *Lhx1* gene locus, which also displayed increased H3K4-trimethylation marks (Figure 2(h, k, l)). As a positive control, we included *MyoD* as muscle-specific gene, which is usually marked by repressive H3K27 trimethylation in neurons [42]. Together, the targeted ChIP experiments presented here suggest that DNMT1 regulates *Lhx1* expression by promoting the establishment of repressive H3K27me3 and the removal of permissive H3K4me3 signatures at regulatory *Lhx1* gene regions. This is in part reminiscent to what we found for the DNMT1-dependent regulation of *Pak6* expression, which is mediated by the concerted action of DNMT1 and EZH2, the core enzyme of the PRC2, promoting the setup of repressive H3K27me3 marks in regulatory regions of the *Pak6* locus [12].

### *DNMT1-mediated alterations in histone acetylation contribute to the modulation of* Lhx1 *expression*

**Figure 3.**
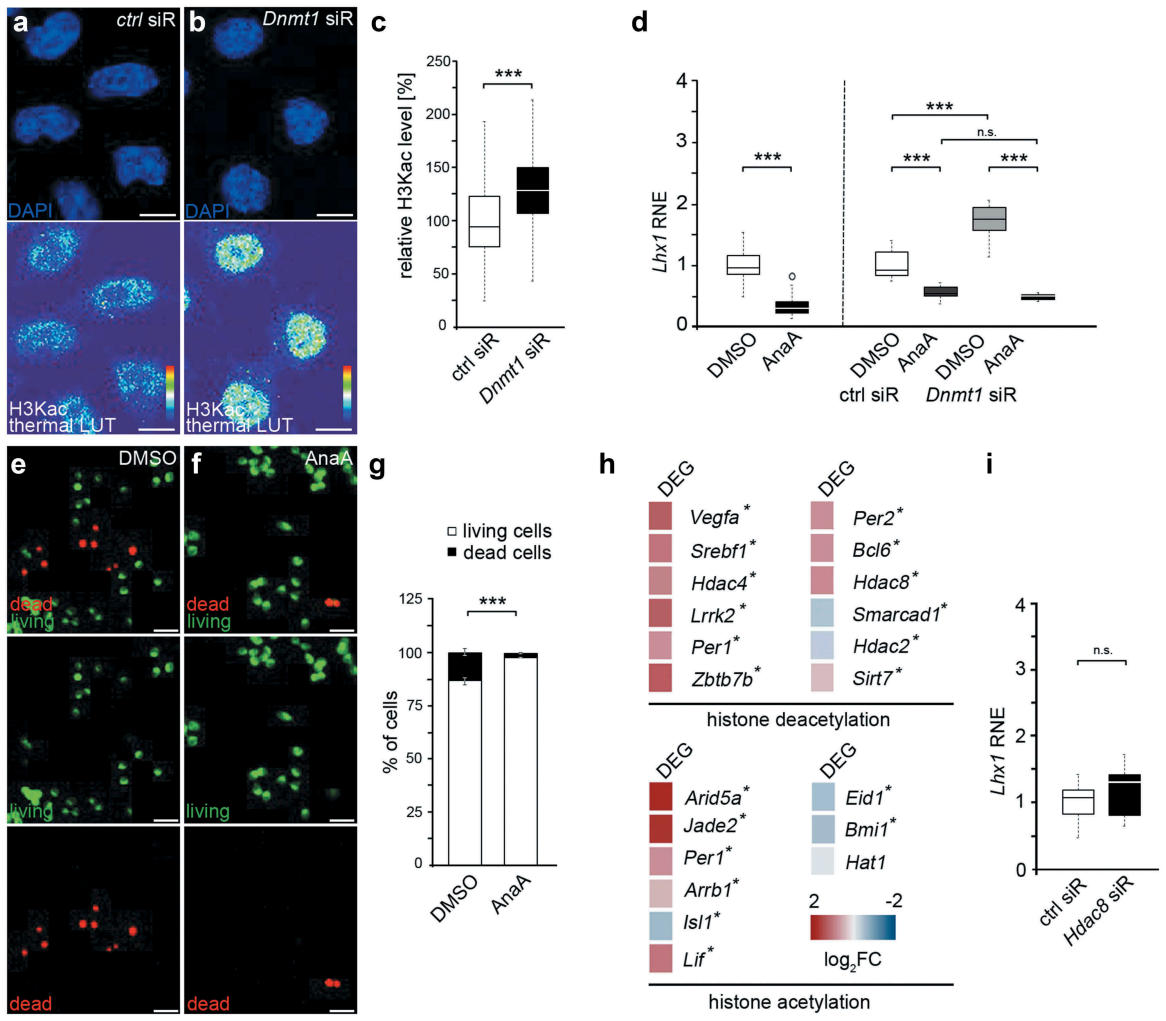
*Lhx1* transcription is modulated by DNMT1-dependent histone acetylation and deacetylation processes

Besides affecting H3K27 and H3K4 trimethylation [5–8,12], DNMT1 was further reported to act on histone acetylation, which leads to open chromatin [43,44]. In line with this, we detected significantly increased H3K9/K14/K18/K23/K27 acetylation levels in *Dnmt1* siRNA-treated N2a cells compared to controls by performing immunocytochemistry with a pan-specific antibody (Figure 3(a–c)). This indicates that DNMT1 can also modulate transcription by negatively acting on permissive histone acetylation marks. The following set of experiments was designed to address whether the DNMT1-mediated modulation of histone acetylation contributes to the transcriptional regulation of *Lhx1* as well as its cellular effects.

(a–c) Representative microphotograph of control (a) and *Dnmt1* siRNA-treated (b) N2a cells stained for H3K9/14/18/23/27ac, shown as thermal colour code indicating fluorescence intensity (thermal LUT) and nucleus staining (DAPI). The mean grey value is analysed and normalized to control siRNA (c, n = 540 cells for each condition). (d) *Lhx1* expression levels in N2a cells treated either with DMSO, anacardic acid (left), control siRNA and DMSO together, control siRNA and anacardic acid in combination, *Dnmt1* siRNA in combination with DMSO and *Dnmt1* siRNA together with anacardic acid (right). Student’s *t*-test was applied for the comparison shown in the left part of the diagram. Two-way ANOVA and Tukey Test were performed for the analysis depicted in the right part of the diagram. The two-way ANOVA revealed that the siRNA conditions, the (inhibitor) treatment conditions as well as the combination of both were highly significant (***P < 0.001). The significances resulting from the post-hoc Tukey Test are indicated in the diagram. (e–g) Representative microphotographs of N2a cells treated with DMSO or anacardic acid and stained for living (green) and dead cells (red) analysed as percentage of total cell number (g). (h) Heat-map of differential expression levels for genes associated with GO terms histone deacetylation and acetylation in FAC-sorted E16 control and *Dnmt1*-deficient POA cells revealed by RNA sequencing (*DEG with p < 0.05, Bonferroni-corrected). (i) *Lhx1* expression in *Hdac8* siRNA-treated N2a cells compared to control cells (g) and Scale bar: 10 µm in (a) and (b), 40 µm in (e) and (f). ***P < 0.001; Student’s *t*-test. AnaA, anacardic acid; Ctrl, control; RNE, relative normalized expression.

First, we examined whether histone acetylation affects *Lhx1* expression by treating N2a cells with the histone acetyltransferase inhibitor anacardic acid, causing global histone deacetylation [45,46]. Inhibiting histone acetylation resulted in significantly decreased *Lhx1* expression levels compared to DMSO control treatment (Figure 3(d), left side of the diagram). To investigate whether DNMT1 controls *Lhx1* expression by modulating histone acetylation, we determined whether the elevated *Lhx1* expression levels seen upon *Dnmt1* siRNA treatment (Figures 1(d) and 3(d)-right side of the diagram) can be reversed by concurrent application of the histone acetyltransferase inhibitor anacardic acid. Indeed, collective administration of anacardic acid together with *Dnmt1* siRNA reduced the boosted *Lhx1* transcription levels seen after *Dnmt1* depletion to levels which were even below the *Lhx1* expression levels of the control treatment (control siRNA and DMSO; Figure 3(d)). This proposes that (i) permissive histone acetylation promotes *Lhx1* expression and that (ii) DNMT1 represses *Lhx1* in part by impeding the establishment of such permissive histone acetylation marks.

We have previously characterized the relevance of DNMT1 and LHX1 function for survival regulation in POA-derived cortical interneurons [26,27]. We identified several downstream targets of LHX1, through which cell survival in immature POA-derived cortical interneurons is controlled. We showed that LHX1 drives the expression of pro-apoptotic genes and negatively acts on the transcription of pro-survival genes [26]. Hence, tight orchestration of *Lhx1* expression during interneuron development is required to maintain the delicate balance of interneuron cell death and survival, and hence the control over correct interneuron numbers, for which we propose DNMT1 as up-stream repressor. As we here provided evidence that the DNMT1-mediated repression of *Lhx1* is in part achieved by impeding the establishment of permissive histone acetylation marks (Figure 3(a–d)), we next investigated whether manipulating histone acetylation affects cell survival regulation. For this, we determined cell death rates of N2a cells that were treated with the histone acetylation inhibitor anacardic acid by applying a live/dead assay. We indeed detected elevated proportions of living cells in anacardic acid-treated samples (Figure 3(e–g)). This is in line with the diminished *Lhx1* levels observed upon anacardic acid treatment (Figure 3(d)), and the role of LHX1 in promoting cell death, as we previously reported [26].

Our data so far indicate that DNMT1-mediated modulation of histone acetylation could contribute to *Lhx1* transcriptional control with potential implications for cortical interneuron migration and survival. In support of this, numerous studies propose a DNMT1-dependent transcriptional regulation of genes encoding for histone-modifying complexes as a potential mechanism for a crosstalk of DNMT1 with histone modifications [9–12]. Such transcriptional control could also affect the concerted actions of histone acetylases (HATs) and histone deacetylases (HDACs), and hence the balance of histone acetylation and deacetylation, respectively [47]. Indeed, by screening the RNA sequencing dataset of FACS-enriched embryonic (E16) wild-type and *Dnmt1*-deficient POA cells [27], we found evidence that DNMT1 regulates the expression of genes associated with histone deacetylation. In *Dnmt1*-deficient POA cells we observed significant changes in the expression of *Hdac2, Hdac4* and *Hdac8* encoding histone deacetylases (Figure 3(h)). In addition, numerous other genes associated with histone deacetylation were changed in expression upon *Dnmt1* deletion. While the expression of *Hat1* encoding for a histone acetylase was not significantly altered, the expression levels from other histone acetylation-related genes like *Arid5a* [48] and *Jade2* [49] were changed prominently in *Dnmt1*-deficient POA cells compared to controls (Figure 3(h)). Together, these transcriptional alterations induced by *Dnmt1* deletion support a role of DNMT1 in the transcriptional regulation of genes related to histone acetylation/deacetylation complexes.

HDAC8 was already described as a potent inhibitor of *Lhx1* transcription in cranial neural crest cells [50] and is implicated in cell survival regulation [51]. However, *Hdac8* expression was significantly increased in FACS-enriched *Dnmt1-*deficient POA cells (E16) (Figure 3(h)). While being consistent with a repressive function of DNMT1, this does not provide a logic explanation for the elevated pan-histone acetylation induced by *Dnmt1* siRNA in the N2a cell culture model, as augmented HDAC8 expression would rather be consistent with diminished histone acetylation. Moreover, *Lhx1* expression levels were not changed in *Hdac8* siRNA-treated N2a cells (Figure 3(i)), indicating that HDAC8 is not affecting *Lhx1* transcription in our cell culture model.

*Hdac2* was the only histone deacetylase-encoding gene, which we found diminished in *Dnmt1*-deficient POA cells, indicating that DNMT1 directly or indirectly promotes its expression (Figure 3(h)). HDAC2 was already reported to regulate the expression of *Lhx1* [52]. Hence, a DNMT1-dependent promotion of histone deacetylation by enhancing *Hdac2* expression represents a potential scenario for the DNMT1-mediated transcriptional repression of *Lhx1*. Whereas deciphering the underlying mechanism requires further investigation, our data so far indicate that DNMT1-mediated gene expression regulation in immature interneurons could be realized via the modulation of histone acetylation in addition to the previously reported crosstalk with histone methylation.

## Discussion

The development of interneurons, including their long-range migration and their subtype-specific differentiation as well as their survival, is strictly controlled by various regulatory mechanisms [24]. Here we provided evidence that DNMT1 regulates the expression of the LIM homeodomain transcription factor LHX1, a relevant regulator of cortical interneuron development. *Lhx1* was shown to be expressed in a proportion of POA interneurons during brain development, promoting proper migration from their subpallial origin towards cortical targets as well as controlling their survival [26]. Our current data support a role of DNMT1 in executing transcriptional control over *Lhx1* through DNA methylation-independent mechanisms. Apart from DNMT1-dependent modulation of H3K4 and H3K27 trimethylation, partially organized in bivalent regions, DNMT1-mediated interference with histone acetylation may contribute to the regulation of *Lhx1* gene activity in interneurons and neuron-like cells.

Epigenetic mechanisms are key for neuronal development and function and are implicated in diverse neurological diseases and psychiatric disorders [13,14]. DNA methylation exerted by DNA methyltransferases (DNMTs) was shown to play a major role in the regulation of gene transcription and the modulation of neuronal differentiation programs [53–55]. Thereby, DNA methylation was often reported to silence gene transcription by preventing the binding of transcription factors to the DNA [53–55]. We recently reported that the function of DNMT1 is fundamental for the maturation of POA-derived cortical interneurons [27]. Interestingly, we observed that the majority of genes in post-mitotic POA interneurons which revealed elevated expression upon *Dnmt1*-deletion also showed increased methylation states. This is not in line with the proposed model of repressive DNMT1-dependent DNA methylation. Recently studies added new aspects on how DNA methylation or DNMTs may contribute to the control of gene activity. These include a crosstalk with histone tail modifications or RNA silencing that concertedly contribute to the complex network of gene regulation [53,56]. For this reason, we asked whether the regulation of relevant DNMT1 downstream target genes depends on non-canonical DNMT1 functions. In this context, we recently showed that the expression of the gene coding for the serine/threonine-protein kinase PAK6, which regulates interneuron morphology and survival [12,27], is modulated by DNMT1-dependent establishment of repressive H3K27me3 marks at gene promoter sites [12]. Following this line of research, we here asked, whether *Lhx1* is likewise regulated by DNMT1-dependent changes in the histone code, as in contrast to *Dnmt1* deletion-induced alteration in *Lhx1* expression, no respective changes in the DNA methylation levels were found. Our data emphasize a bivalent regulation of *Lhx1* by a DNMT1-dependent modulation of both repressive H3K27me3 and permissive H3K4me3 marks.

An interaction of DNMT1 with enzymes involved in establishing H3K27me3 marks like EZH2 as the main methyltransferase of the polycomb repressor complex 2 (PRC2), as well as a transcriptional regulation of associated genes was already reported for non-neuronal cells in different studies [11,57,58]. We recently revealed that in POA interneurons and neuron-like N2a cells the DNMT1-dependent establishment of H3K27-trimethylation marks relies on protein–protein interactions between DNMT1 and EZH2 [12]. In turn, DNMT1-dependent repression of activating H3K4me3 marks in control cells appears to be achieved via transcriptional control of relevant key players. This assumption was based on the observation that in *Dnmt1*-deficient *Hmx3*-expressing POA cells an enhanced expression of H3K4me3-associated genes was revealed [12,27]. However, other ways of action are likewise conceivable.

A simultaneous association of permissive H3K4me3 and repressive H3K27me3, like we and others observed for the *Lhx1* promoter [59–61], is typically found for many developmental genes adopting a ‘winner-takes-all’ principle [36,38,39,62] with the decision about gene transcription being defined by the proportion of these histone modifications. Such bivalent gene regulation, initially reported for the repression of lineage restricting genes in early embryogenesis (reviewed in [63]), enables the repression of genes until their expression is required. Since LHX1 regulates the survival and migration of specific interneurons from the POA within a given time window [26], a highly coordinated expression of this transcription factor is of great importance. A bivalent regulation of *Lhx1* expression would allow for such a temporally and spatially limited expression, which seems to depend on DNMT1 function.

Besides the connection to histone methylation, DNMT1 also interacts with key enzymes relevant for histone acetylation and deacetylation [43,44]. Acetylated histones are highly associated with euchromatic gene regions and activated gene transcription, while histone deacetylation results in ‘closed’ heterochromatin and gene repression [64,65]. Histone acetylation and deacetylation processes are intimately linked to proper development and function of several cortical interneuron types and enable a dynamic change of gene accessibility [66,67]. The data presented here indicate a DNMT1-dependent repression of activating histone acetylation marks in immature POA interneurons, as well as a regulation of the *Lhx1* expression level by the histone acetylation status. Based on this, we propose the hypothesis that DNMT1 represses *Lhx1* transcription at least partly by contributing to changes in histone acetylation levels. DNMT1 has already been reported to be associated with HDAC activity, which removes histone acetylation marks to silence gene transcription. For example, Fuks *et al*. [43] identified a specific domain in the DNMT1 protein that partially contributes to transcriptional repression by recruiting histone deacetylase activity. Apart from this, DNMT1 was also shown to directly bind HDAC2 and the co-repressor DMAP1 to form a repressive transcription complex [44]. Thus, a DNMT1-dependent removal of acetyl groups from histone tails could account for *Lhx1* repression in POA interneurons and N2a cells. Interestingly, an HDAC-dependent repression of *Lhx1* was already shown in cranial neural crest cells for the class I histone deacetylase HDAC8 [50], in which the enzyme prevents the aberrant expression of homeobox transcription factors during skull development. For the cell types investigated here, HDAC8, in turn, seems of subordinate relevance for the transcriptional control of *Lhx1*, as *Hdac8* siRNA application had no effect on *Lhx1* transcription levels. This underlines that the function of HDACs appears to be cell type specific and likely depend on cell-specific cofactors and their integration into protein complexes. Apart from that, the regulation of *Lhx1* expression was also described for the class I histone deacetylases HDAC1 and HDAC2 in non-neuronal progenitor cells [52]. Here, we detected reduced levels of *Hdac2* expression in *Dnmt1*-deficient POA cells, which is in line with the increased pan-histone acetylation determined upon *Dnmt1* depletion. Consequently, a DNMT1-dependent transcriptional regulation of HDAC2 could represent a possible scenario for how DNMT1 modulates *Lhx1* transcription through a crosstalk with histone acetylation. Moreover, we identified numerous transcripts related to histone acetylation and deacetylation that were altered in expression in *Dnmt1*-deficient POA cells, indicating multiple levels of regulation. Besides, other mechanisms that would enable a crosstalk between DNMT1 and histone acetylation, for example, via transcriptional control over long non-coding RNA expression that in turn can recruit or avoid the binding of chromatin-modifying complexes [68], or an interaction of DNMT1 with the histone acetylation machinery at protein level similar to what we identified for the DNMT1-dependent establishment of H3K27me3 marks [12], are likewise conceivable and subject of further investigations.

Furthermore, since the regulation of gene expression through epigenetic mechanisms is based on a complex network of diverse factors that regulate different histone tail modifications, we cannot rule out that additional mechanisms like histone phosphorylation, ubiquitination, or deimination contribute to the DNMT1-dependent regulation of *Lhx1* expression. We likewise cannot exclude that the effects we described could also partially represent secondary effects through intermediary factors, and not necessarily be due to the direct interaction of DNMT1 with histone methylating complexes. For this, the identification of potential binding partners and whole protein complexes interacting with DNMT1 is of great relevance to fully understand the complex crosstalk of bivalent gene sites by histone 3 trimethylation and histone acetylation.

Together, the transcriptional control by epigenetic mechanisms once more emerges as a complex interplay of numerous factors, likely acting in large complexes that integrate intracellular and extracellular cues to drive cell differentiation and maturation processes.

## Methods

### Animals

For all experiments, transgenic mice on C57BL/6J background were used including *Hmx3-Cre/tdTomato/Dnmt1* wild-type as well as *Hmx3-Cre/tdTomato/Dnmt1 loxP^2^* mice. Transgenic mice were generated by crossing *Hmx3-Cre* mice (obtained from Oscar Marin, King’s College, London, UK and described in Gelman *et al*. [25]) with *tdTomato* transgenic reporter mice (obtained from Christian Hübner, University Hospital Jena, Germany and described in Madisen et al. [69]) and *Dnmt1 LoxP^2^* mice (*B6; 129Sv-Dnmt1tm4Jae/J*, Jaenisch laboratory, Whitehead Institute, USA). *Cre*-mediated deletion in *Dnmt1* mice leads to out-of-frame splicing from exon 3 to exon 6, resulting in a *Dnmt1* null allele [70]. Transgenic mice are abbreviated as *Dnmt1* WT and *Dnmt1* KO in text and figures. Mice were housed under 12 h light/dark conditions with *ad libitum* access to food and water. All animal procedures were approved by the local government (Thueringer Landesamt, Bad Langensalza, Germany) and performed in strict compliance with the EU directives 86/609/EWG and 2007/526/EG guidelines for animal experiments. Study design and experiments were performed according to the ARRIVE guidelines.

### Preparation of POA single cells

For the preparation of cells of the embryonic preoptic area (POA), timed pregnant *Dnmt1* WT and KO mice were killed by an intraperitoneal injection of 1x PBS (pH 7.4) with 2.5 μg chloral hydrate per g body weight. Embryonic POA was prepared under visual control, dissociated with 0.04% trypsin (Thermo Fisher Scientific) in Hank´s balanced salt solution (Invitrogen) for 17 min at 37 °C prior trituration and removal of cell aggregates by filtering through a 200 µm nylon gauze. Preparations of *Cre*-positive embryos were used for fluorescence-activated cell sorting. POA cells of *Cre*-negative embryos were used for morphometric studies. They were seeded at densities of 300 cells/mm^2^ on coverslips (Ø 12 mm) coated with 19 µg/µL laminin (Sigma-Aldrich) and 5 µg/µL poly-L-lysine (Sigma-Aldrich) and cultured according to Symmank et al. [12] at 37 °C and 5% CO_2_.

### Fluorescence-activated cell sorting (FACS) of POA cells

FACS of *tdTomato* reporter-positive cells was performed as described in Pensold et al. [27]. FACS-enriched cell pellets were either frozen directly for DNA isolation or dissociated with TRIzol™ Reagent (Life Technologies) for RNA isolation.

### RNA Sequencing and MeDIP analysis of embryonic POA cells

RNA sequencing and methylated DNA immunoprecipitation (MeDIP) sequencing of FACS-enriched POA cells were described and performed by Pensold et al. [27] and reanalysed in this study. Briefly, pooled samples were tested in technical duplicates for RNA sequencing. Due to higher quantities of required material for MeDIP sequencing, one pooled sample was evaluated per genotype and a special bioinformatic pipeline for computational analysis was applied for such rare samples as previously described in Pensold et al. [27] and Pensold *et al*. [71]. Complete data set of RNA sequencing and MeDIP analysis is provided by Pensold *et al*. [27] and uploaded at GEO with the series number GSE146968. Heat-maps were generated using R package pheatmap (https://CRAN.R-project.org/package=pheatmap). For heat-maps showing a comparison between two datasets, data were normalized to WT and log^2^ fold-change to KO is depicted.

### N2a cell culture

Neuro-2a (N2a) cells were grown in culture medium consisting of DMEM with high glucose, sodium pyruvate (Thermo Fisher) and GlutaMAX (Invitrogen), 10% FBS (Biowest), 100 U/mL penicillin (Gibco), 100 µg/mL streptomycin (Gibco) at 37 °C, 5% CO_2_, and 95% humidity. When reaching 75% confluence, N2a cells were mechanically dissociated and seeded at densities of 100 cells/mm^2^ on coverslips (Ø 12 mm) coated with 19 μg/mL laminin (Sigma-Aldrich) and 10 μg/mL poly-L-lysine (Sigma-Aldrich) in GBSS. For further treatment, N2a cells were incubated in culture medium (DMEM, 10% FBS, 100 U/mL penicillin, 100 µg/mL streptomycin) at 37 °C, 5% CO_2_, and 95% humidity for 24 h.

### Transfection with siRNA and inhibitor treatment

Transfection of N2a cells with siRNA was performed via lipofection using Lipofectamine™ 2000 (Thermo Fisher Scientific), according to the manufacturer’s protocol and as described in Zimmer *et al*. [72] and Pensold et al. [71]. Mouse *Dnmt1* siRNA oligos (30 nM; siRNA sc-35203, Santa Cruz Biotechnology), *Hdac8* siRNA oligos (30 nM; siRNA sc-35548, Santa Cruz Biotechnology), or control siRNA (15 nM; BLOCK-iT Alexa Fluor red/green fluorescent oligo, Invitrogen) were applied for 5 h in antibiotic- and serum-free Opti-MEM (Thermo Fisher Scientific). Afterwards, cells were grown in culture medium (DMEM with high glucose, sodium pyruvate and GlutaMAX, 10% FBS, 100 U/mL penicillin, 100 µg/mL streptomycin) for 24 h at 37 °C, 5% CO_2_, and 95% humidity if not stated differently.

N-phthalyl-L-tryptophan (20 μM, RG108, Sigma-Aldrich) was used to block DNA methylation. To inhibit histone methyltransferases, N2a cells were treated with 100 nM of 3-deazaneplanocin A (DZNep; Sigma-Aldrich) and POA cells with 1.5 µM DZNep. Anacardic acid of 40 µM (AnaA, Merck) was used to inhibit histone acetyltransferase function in N2a cells. All inhibitor treatments were performed in culture medium for 24 h at 37 °C, 5% CO_2_, and 95% humidity and as control, N2a cells were treated with dimethyl sulphoxide (DMSO).

### Chromatin-immunoprecipitation and quantitative PCR

For chromatin immunoprecipitation (ChIP), 75% confluent N2a cells were transfected with siRNA in 10 cm well plates. 24 h after transfection, crosslinking of DNA and protein was performed with 1% formaldehyde in PBS. Cells were harvested and aliquoted with a cell number of 1 × 10^6^ cells per tube and centrifuged for 5 min at 1000 x g at 4°C. ChIP was performed as described in Symmank et al. [12] using the following ChIP-valuated antibodies for precipitation: rabbit anti-H3K4me3 (Abcam), rabbit anti-H3K27me3 (Millipore), and rabbit anti-IgG (Abcam). One per cent input control was taken after DNA fragmentation. Protein-DNA cross-linking of probes and input control were removed for 4 h at 65 °C with 200 mM NaOH. Finally, proteins were digested for 1 h at 45 °C in 10 mM EDTA, 40 mM Tris-HCl (pH 6.5) und 20 μg/μl proteinase K (Merck). DNA was precipitated with standard phenol-chloroform isoamyl alcohol (25:24:1) extraction and purified with the DNA Clean & Concentrator-5 Kit (Zymo Research) according to the manufacturer’s guidelines. To quantitatively asses the amount of specific DNA fragments, primer-specific pre-amplification of probes was performed in the T-gradient PCR Cycler (Bio-Rad) for 20 cycles prior quantitative analysis with the Real-Time PCR-System *qTOWER^3^* (Analytik Jena). For both PCR reactions, Luminaris Color HiGreen qPCR Master Mix (Thermo Fisher Scientific) was used according to the manufacturer’s protocol. Following primer sequences were used (indicated as 5′ → 3′; fw, forward; rev, reverse): *Gapdh* fw AACGACCCCTTCATTGACCT, *Gapdh* rev TGGAAGATGGTGATGGGCTT, *Lhx1*-#1 fw AGACCTCTGATCCGAAGCTG, *Lhx1*-#1 rev AACGACTTCTTCCGGTGAGT, *Lhx1*-#2 fw TGGTCCCTTTGCTCTCCATT, *Lhx1*-#2 rev GGGCGACTCACAGATTTCCT, *Lhx1*-#3 fw GGCAACTGTCTGAATATCATGGT, *Lhx1*-#3 rev TGACAGATTTGCAGGGCTTG, *MyoD* fw CTCACAGAGTCCAGGCCAG, *MyoD* rev TGTTCTGTGTCGCTTAGGGA. Normalization of DNA content was performed according to the per cent-input method in relation to the analysed input probes [73].

### RNA isolation and expression analysis

RNA isolation of FACS-enriched POA cells was performed as described in Pensold *et al*. [27]. For expression analysis of siRNA- or inhibitor-treated N2a cells grown in six well plates, cells were harvested with TRIzol™ Reagent (Thermo Fisher Scientific) following the manufacturer’s guidelines. RNA was isolated using 1-Bromo-3-chloropropane, centrifuged for 30 min at 13,000 × g and 4 °C and the aqueous phase was purified with the RNA Clean & Concentrator-5 kit (Zymo Research) including DNAse treatment according to manufacturer’s protocol. Superscript IV*™* first-strand synthesis system (Thermo Fisher Scientific) was used for cDNA synthesis according to the manufacturer’s instructions with the same amount of input RNA in all compared probes. Quantitative reverse transcription PCR was performed with Luminaris Color HiGreen qPCR Master Mix (Thermo Fisher Scientific) according to manufacturer’s protocols and following primers were used (indicated as 5′ → 3′; fw, forward; rev, reverse): *Lhx1* fw GGAGCGAAGGATGAAACAGC, *Lhx1* rev TGCGGGAAGAAGTCGTAGTT, *Rps29* fw GAAGTTCGGCCAGGGTTCC, *Rps29* rev GAAGCCTATGTCCTTCGCGT. As housekeeping gene, *Rps29* was used. Each sample was tested in three biological replicates analysed in separate qPCR runs with one to three technical replicates. The qPCR program included the following optimized steps: UDG pre-treatment at 50 °C for 2 min, initial denaturation at 95 °C for 10 min, denaturation at 95 °C for 15 sec as well as annealing and elongation at 60 °C for 1 min. Denaturation and annealing/elongation steps were repeated 40 times and primer dimers were excluded by a melting curve analysis. Data were analysed with ΔΔ*Ct* method [74].

### Immunocytochemistry

N2a cells, cultured on coverslips were fixed with 4% PFA/1x PBS for 10 min and immunocytochemistry was performed as previously described in Zimmer *et al., 2011* [72]. A pan-specific rabbit-anti-H3K9/K14/K23/K27 acetylation (Abcam) was used as the primary antibody. As secondary antibody, Cy3-goat anti-rabbit IgG (1:1000; Jackson Laboratory) was used. For analysis of cell morphology, incubation with Alexa Fluor™ 488 Phalloidin (Thermo Fisher Scientific) was performed according to the manufacturer’s guidelines.

### Live-dead-assay

N2a cells cultured on coverslips were stained after inhibitor treatment for living and dead cells with the LIVE/DEAD™ Cell Vitality Assay Kit for mammalian cells (Invitrogen) according to the manufacturer’s protocol.

### Migration assay with N2a cells

Standardized imaging plates (Eppendorf; 170 µm glass thickness) were coated with matrigel (GelTrex™; Thermo Fisher) according to the manufacturer’s instructions using a working concentration of 0.1 mg GelTrex^TM^ diluted in 1 mL N2a culture medium. One hundred microlitre matrigel working solution was added per well and incubated for 60 min until hardening of the substrate. N2a cells were seeded with a density of 57 cells/mm^2^ and treated with the inhibitor DZNep after 24 h as described above. To avoid phototoxic effects during imaging, culture medium was exchanged to DMEM without phenol red (Invitrogen) with 10% FBS (Biowest), 100 U/mL penicil-lin (Gibco), 100 µg/mL streptomycin (Gibco). Forty-eight hours after seeding, N2a cells were imaged every 15 min for 20 h at 37 °C and 5% CO_2_.

### Microscopy and data analysis

Fluorescent images were taken with the inverted confocal laser scanning microscope *TCS SP5* (Leica). Life cell imaging of migrating cells and images of the Live-Dead-Assay were taken with the DMi8 with thunder imaging platform (Leica). Photographs were analysed with Fiji (ImageJ) software [75]. Background correction was performed for fluorescence intensity measurement. Mean fluorescent intensity of *Dnmt1* siRNA-treated cells was normalized to control siRNA-treated cells. Quantitative RNA results were analysed by efficiency corrected ΔΔ*Ct* method and presented in relation to control samples. Photoshop CC was applied for image illustration. Significance was analysed with two-tailed *Student’s t*-test or two-way ANOVA with Tukey Test. Shapiro–Wilk was used as Normality Test. Significance levels: P value <0.05 *; P value <0.01 **; P value <0.001 ***. If not stated differently, experiments were repeated three times.

## Data Availability

The reanalyzed RNA sequencing and MeDIP data sets comparing *Hmx3-Cre/tdTomato/Dnmt1* wild-type as well as *Hmx3-Cre/tdTomato/Dnmt1 loxP^2^* mice that are used in this study are published by Pensold *et al*. (27) and uploaded at GEO with the series number GSE146968.
